# Cytotoxicity and Genotoxicity of Epoxy Resin-Based Root Canal Sealers before and after Setting Procedures

**DOI:** 10.3390/life12060847

**Published:** 2022-06-07

**Authors:** Mijoo Kim, Marc Hayashi, Bo Yu, Thomas K. Lee, Reuben H. Kim, Deuk-won Jo

**Affiliations:** 1 Restorative Materials and Applied Dental Research Laboratory, UCLA School of Dentistry, Los Angeles, CA 90095, USA; vagusmj@gmail.com (M.K.); mhayashi@dentistry.ucla.edu (M.H.); boyu@dentistry.ucla.edu (B.Y.); tlee@dentistry.ucla.edu (T.K.L.); rkim@dentistry.ucla.edu (R.H.K.); 2 Section of Restorative Dentistry, UCLA School of Dentistry, Los Angeles, CA 90095, USA; 3 Department of Prosthodontics, Section of Dentistry, Seoul National University Bundang Hospital, Seongnam 13620, Korea

**Keywords:** biocompatibility, comet assay, cytotoxicity, genotoxicity, resin-based sealer, root canal treatment, risk assessment

## Abstract

Epoxy resin-based sealers are commonly used for successful endodontic treatment. This study aimed to evaluate the cytotoxicity and genotoxicity of epoxy resin-based sealers under unset and set conditions. Three epoxy resin-based sealers were used: Adseal, AH Plus, and Dia-Proseal. To test cytotoxicity, an agar overlay test and a 3-[4,5-dimethylthiazol-2-yl]-2,5 diphenyl tetrazolium bromide (MTT) assay were performed using unset and set sealers on L929 mouse fibroblasts. The genotoxicity test of the comet assay was performed using the same cell line. Extract dilutions in the culture media were used as test materials for the MTT and comet assays. The comet tail produced by the damaged DNA was calculated by image analyses. Statistical analyses were performed using one-way analysis of variance and Tukey’s post hoc test. Unset sealers did not show defined decolorized areas. Hardened specimens of resin-based sealers showed circular discolored zones in the agar overlay test. Dia-Proseal was the least cytotoxic after hardening. These results were confirmed in the MTT assay. Cell viability was significantly higher in cells treated with hardened sealers in both groups than that in cells treated with freshly mixed sealers in the MTT assay. Unset AH Plus^®^ and Dia-Proseal™ significantly increased cell viability with decreasing dilution. Adseal™ was the least cytotoxic. Freshly mixed Adseal™ was more genotoxic when freshly mixed than when set. Unset epoxy resin-based sealers were generally more cytotoxic and genotoxic than set materials. Cytotoxicity does not always match the genotoxicity results; therefore, various test tools are required to test toxicity. It is necessary to properly evaluate the toxic effects to establish a biocompatibility test that mimics clinical conditions.

## 1. Introduction

Endodontic treatments include a sequence of procedures for the removal of the infected pulp to eliminate infection and protect the decontaminated tooth from future microbial invasion. Access opening, removal of the pulp tissue, shaping with irrigation, and filling procedures are the basic endodontic processes. Root canal filling is performed for permanent obturation and to provide support to the circumferential restorations. An ideal root canal filling material is biocompatible and has antimicrobial properties, enabling significantly longer retention of the tooth. In addition, materials that fill root canals should provide an effective seal and be dimensionally stable, with the ability to induce biological cell responses that contribute to regeneration [1]. Based on these requirements, dental companies and researchers have sought to develop innovative endodontic filling materials for successful root canal treatment.

Root canal sealer is a primary factor for a successful canal filling process, which fills the space between the inner root surface and artificial filling materials (generally gutta-percha cone) after pulp removal, canal enlargement, and irrigation. Root canal sealers may also kill the bacteria remaining in the accessory canals that are not removed during irrigation. Currently, many types of endodontic sealers, including materials such as zinc oxide eugenol, epoxy resin, glass ionomer, calcium hydroxide, and mineral trioxide aggregate, are available commercially. Sealing ability, low solubility, dimensional stability, and good biocompatibility are required regardless of the sealer type [2]. Resin-based sealers possess acceptable physical and biological properties [3]. Epoxy resin-based sealers have been introduced in endodontic practice because of their favorable characteristics, such as adhesion to the tooth structure, long working time, ease of mixing, and good sealing ability [4,5]. Epoxy resin-based sealers undergo hardening by chemical reactions after mixing the constituents and are stable root canal sealants.

According to International Organization for Standardization (ISO) 1942, biocompatible materials used in dentistry do not cause adverse or unwanted side effects when they come into contact with the living environment. The biological effects of epoxy resin-based sealers, when used in the oral environment, must be evaluated before their release into the market. The experimental conditions for the evaluation simulate the clinical application of dental materials following ISO 7405. Biocompatibility tests conducted to evaluate the biocompatibility of medical devices used in dentistry should use the product in their “as-used state.” However, many investigators have disregarded that in clinical usage, and freshly mixed sealers may unintentionally penetrate the root apex and come in contact with the nearby tissues for a few minutes to hours prior to hardening. Furthermore, most biocompatibility and toxicity tests for endodontic sealers have used diluted extracts obtained from preset sealers [6,7,8,9,10], which is not in accordance with clinical conditions.

Root canal sealers may contact the root apex within their setting time and can be influenced by body fluids and blood flow. Although sealers are designed to remain inside the canal, substances that are frequently extruded into the periradicular area through the apical foramen and lateral and accessory canals result in negative biological effects [11,12,13]. They may temporarily or permanently enter the bloodstream or contact other tissue fluids, causing irritation, inflammation, and possibly delayed wound healing after endodontic procedures [14,15]. The setting time for epoxy resin-based root canal sealers depends on the products and usually takes a few hours to days before fully hardening in vivo [16]. During that time, root canal sealers may cause adverse local and/or systemic effects on periradicular tissues and alveolar bone, attributable to the release of extractable monomers and/or other inorganic and organic ingredients that can degenerate the tissue underneath the endodontic sealer [17]. Therefore, an experimental system that reflects clinical conditions is necessary to fully determine the toxicity of dental materials in the human body.

This study aimed to evaluate the biocompatibility of freshly mixed and hardened epoxy resin-based sealers, considering their clinical application. We evaluated the cytotoxicity and genotoxicity of unset and hardened sealers using agar overlay, 3-[4,5-dimethylthiazol-2-yl]-2,5 diphenyl tetrazolium bromide (MTT), and comet assays.

## 2. Materials and Methods

### 2.1. Cell Preparation

L929 mouse fibroblasts (Korean Cell Line Bank, Seoul, Korea) were cultured in Roswell Park Memorial Institute (RPMI) 1640 growth medium supplemented with 10% fetal bovine serum, 150 IU/mL penicillin, 150 μg/mL streptomycin, 0.125 μg/mL amphotericin B, and 0.1 mg/mL geneticin (all from Gibco, Grand Island, NE, USA).

### 2.2. Test Materials

Three epoxy resin sealers were prepared for this test: Adseal™ (Metabiomed, Cheongju-si, Korea), AH Plus^®^ (Dentsply, Charlotte, NC, USA), and Dia-Proseal™ (DiaDent, Cheongju-si, Korea). Table 1 shows their detailed information. These resins were used freshly prepared or after hardening.

### 2.3. Sample Preparation for the Agar Overlay Test

A Teflon ring (5 mm inner diameter and 2 mm height) was placed on solidified agar before inserting the freshly mixed (unset) sealers. The two components of each sealer and the mixtures were placed on a Teflon ring. When testing hardened sealers, each mixed material was flushed with the rim and allowed to set in a water bath (37 ± 2 °C and 90 ± 10% relative humidity) for 24 h before conducting the test. These were also placed on solidified agar.

### 2.4. Extract Preparations for Other Experiments

Freshly mixed or hardened root canal sealers were prepared using Teflon rings. Sealer extracts were prepared in a cell culture medium using a surface-area-to-volume ratio of approximately 150 mm^2^/mL between the surface of the samples and the volume of the medium, as described in ISO 10993-5.

### 2.5. Agar Overlay Test

The agar overlay test was based on ISO 7405. The cells were cultured until they reached the end of the logarithmic growth phase. Ten milliliters of cell suspension (2.5 × 10^5^ cells/mL) was pipetted into each 100 mm cell culture dish at 37 ± 2 °C in a water-saturated atmosphere with 5% (volume fraction) carbon dioxide for 24 h. Sterile agar was heated to 100 °C and then cooled to 48 °C. Freshly prepared agar and double-concentrated culture medium were mixed and heated to 48 °C. The liquid culture medium from each culture dish was aspirated and replaced with 10 mL of freshly prepared agar/culture medium mixture. The medium mixture was solidified at room temperature for 30 min. Ten milliliters of neutral red solution was added, and the mixture was maintained under dark conditions for 20 min. The excess neutral red solution was removed, and the negative, positive, and experimental samples were placed in each dish with the adjacent samples separated by more than 20 mm. The cell culture dishes with samples were incubated in a cell culture incubator for 24 h. Each test material was independently applied four times before and after the setting procedure. The decolorized zone around the test materials and controls was assessed using an inverted microscope with a calibrated screen, and decolorization and lysis indices for each test sample were determined according to the standard criteria. The cell response was based on the median decolorization and lysis indices from the four replicate tests. The cell response was graded separately for each parameter according to ISO 7405. Natural latex rubber was used as a positive control, and a polyethylene film was used as a negative control, with the same contact area as the test materials. The tests were performed five times independently, with three samples used in each trial.

### 2.6. 3-[4,5-Dimethylthiazol-2-yl]-2,5 Diphenyl Tetrazolium Bromide (MTT) Assay

The cells were seeded at a density of 1 × 10^4^ cells/well in a 24-well plate. After 24 h of adhesion, the prepared extracts were diluted in RPMI 1640 medium and added to each well. Cell viability in each group was determined using the MTT assay after culturing for 3 days. The optical density was measured using a microplate absorbance reader at 570 nm (Epoch; BioTek, Winooski, VT, USA). Phenol and distilled water were used as the positive and negative controls, respectively. Five independent tests were performed with ten samples each. 

### 2.7. Comet Assay of Affected Cells by Root Canal Sealers

The comet assay is a single-cell gel electrophoresis assay used to evaluate cellular DNA damage using specific materials. Dilutions extracted from unset and set sealers determined by the MTT assay were treated with cell lines for 24 h. Subsequently, individual cells were mixed with molten agarose before being applied to slides. These embedded cells were then treated with a lysis buffer and an alkaline solution that relaxes and denatures DNA. Finally, the samples were electrophoresed in a horizontal chamber to separate the intact DNA from the damaged fragments. Following electrophoresis, the samples were dried, stained with a DNA dye, and visualized by epifluorescence microscopy. The damaged DNA (containing cleaved DNA and strand breaks) migrates further than intact DNA and produces a “comet tail”. Image analysis was performed, and the following parameters were calculated:

 Tail DNA % = 100 × tail DNA intensity/cell DNA intensity

Olive tail moment = tail DNA % × tail moment length *

* The tail moment length was measured from the center of the head to the center of the tail.

Five independent tests were performed with five samples in every round.

### 2.8. Statistical Analyses

Statistical analyses were performed using one-way analysis of variance and Tukey’s post hoc test. The significance level was set at α = 0.05. SPSS PASW version 26.0 (SPSS Inc., Chicago, IL, USA) was used for the statistical analyses.

## 3. Results

### 3.1. Cytotoxicity by the Agar Overlay Test

The results of the agar overlay test are shown in Table 2. The decolorized areas from unset sealers inserted in neutral red-stained L929 cells were not defined; the hardened specimens of resin-based sealers showed circular discolored zones. Dia-Proseal™ demonstrated the smallest affected zone size among the set groups (0.04 ± 0.08 cm). The lysis indexes of Adseal™ and Dia-Proseal™ were lower for the set samples than that for the unset samples. Dia-Proseal™ was the least cytotoxic after hardening, with only mild cytotoxicity. Hardened AH sealer^®^ had a smaller zone size than that of the unset one; however, both samples showed severe cytotoxicity.

### 3.2. Cytotoxicity by the MTT Assay

**Figure 1:**
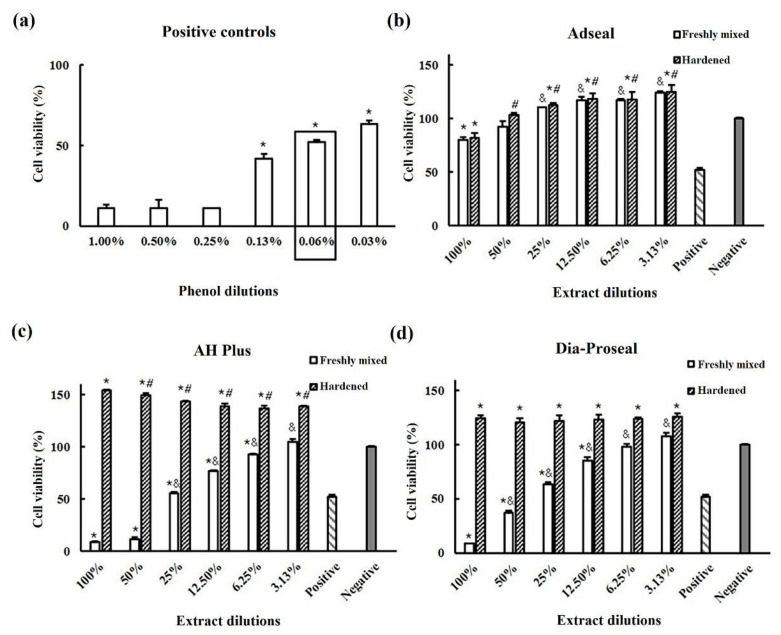
Cytotoxicity test using the 3-[4,5-dimethylthiazol-2-yl]-2,5 diphenyl tetrazolium bromide assay for three epoxy resin-based sealers. (**a**) The phenol concentration is used as a positive control. (**b**–**d**) Cell viability of Adseal™, AH Plus^®^, and Dia-Proseal™ at each dilution, respectively. Positive control, 0.06% phenol; negative control, distilled water. Data are representative of three independent experiments. * Statistically significant differences compared with the negative control for freshly mixed sealers (*p* < 0.05). ^&^ Statistically significant differences compared with cell viability of 100% extracts from freshly mixed sealers. ^#^ Statistically significant differences compared with cell viability of 100% extracts from hardened sealers (*p* < 0.05).

Quantitative evaluations by the MTT assay demonstrated that unset AH Plus^®^ and Dia-Proseal™ significantly increased cell viability with decreasing dilutions (Figure 1c,d, *p* < 0.05). Moreover, the viability of the cells was significantly higher in cells treated with hardened sealers in both groups than that in cells treated with freshly mixed sealers (*p* < 0.05). In the AH Plus^®^ group, the viability of the cells treated with hardened samples (diluted 100 and 50%) was higher than that of cells treated with other sealers (*p* < 0.05). The viability of all AH Plus^®^ and Dia-Proseal™ samples was higher than that of the negative control (*p* < 0.05). The results from the treatment with freshly mixed and hardened Adseal™ samples were similar, regardless of the dilution (Figure 1b).

### 3.3. Genotoxicity by the Comet Assay

**Figure 2:**
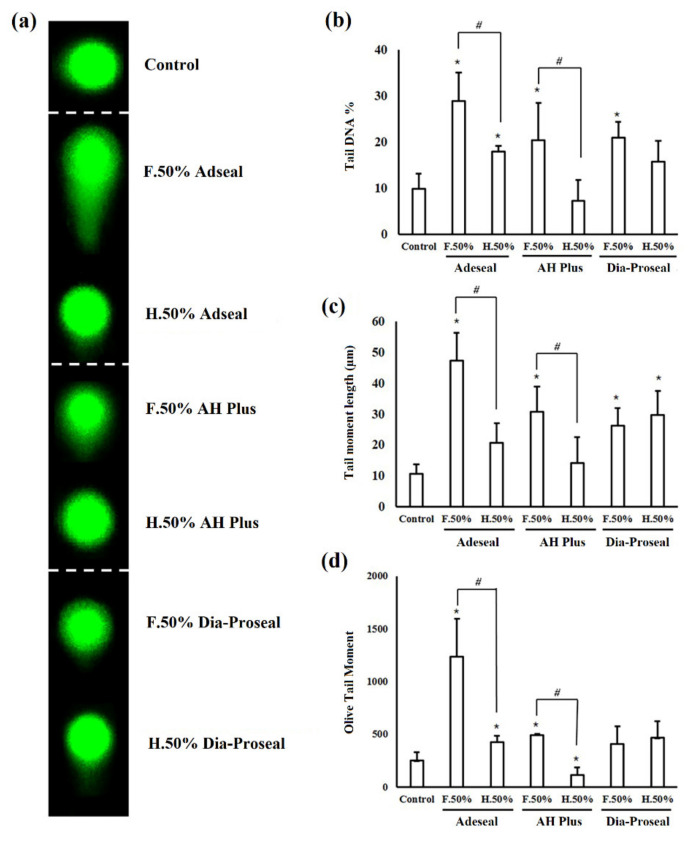
Genotoxicity of the three epoxy resin-based sealers as determined by the comet assay. (**a**) Representative photos of the comet assay are shown (×100, alkaline electrophoresis conditions, 33 V/300 mA for 15 min). (**b**) Tail DNA %, (**c**) tail length, and (**d**) olive tail moment analyzing comet assay images are graphically shown. Data are representative of three independent experiments. * Statistically significant differences compared with the control (*p* < 0.05). ^#^ Statistically significant differences between freshly mixed and hardened sealers (*p* < 0.05). F, freshly prepared; H, hardened.

As shown in Figure 2, the genotoxicity of the epoxy resin-based sealers was measured using the comet assay. Cell images showing increasing levels of DNA damage, as determined by the comet assay, are shown in Figure 2a. DNA damage attributable to freshly mixed Adseal™ and AH Plus^®^ was significantly higher than that of the hardened samples, as shown in Figure 2d (*p* < 0.05). Dia-Proseal™ treatment did not significantly increase genotoxicity, regardless of whether it was freshly mixed or hardened. Among the sealers tested, treatment with freshly mixed Adseal resulted in the highest genotoxicity, as determined by the comet assay images and olive tail moment values.

## 4. Discussion

Root canal sealers play a critical role in successful endodontic treatment. Endodontic sealers and gutta-percha cones form a structure inside the canal that supports tooth movement and endures external forces without any distortion or separation. Most intracanal spaces are occupied by gutta-percha cones, and a small amount of sealer is used to fill the remaining intracanal spaces. However, sealers act as a bridge between the gutta-percha and tooth by tightly occluding the space and protecting the tooth from bacterial contamination, which enables a stable endodontically treated tooth that survives for several years.

This study was conducted using self-curing epoxy resin-based sealers, which are commonly used by clinicians because of their ease of application and long-term availability. These consist of base and catalyst components. The in vitro setting times for the sealers varied from 50 min to 8 h, as shown in Table 1. Adseal™ has a short setting time [18]; therefore, it resulted in high cell viability, regardless of whether it was set or unset. This is consistent with the test results of previous studies [19,20,21]. The relationship between sealer setting time and cytotoxicity has been investigated previously [22,23,24]. When unset samples from AH Plus^®^ and Dia-Proseal™ were in contact with cells for 24 h in the agar overlay test or MTT assay, their components spread into the solidified agar or culture media. Unset sealers resulted in an undefined boundary of decolorized zones and severe cytotoxicity caused by the diluents from the liquid forms in all treatment groups. Interestingly, Adseal™ treatment resulted in a similar viability pattern in the MTT test, regardless of whether it was set or unset (*p* > 0.05); however, it was severely cytotoxic in the agar overlay test. Discrepancies between the agar overlay test and MTT assay demonstrate the different properties of each experiment. Agar overlay tests quantitatively evaluate decolorized zones and lysis indices [25], whereas the MTT assay analyzes the mitochondrial activity of the cells [26]. The use of the agar overlay test for unset sealers is considered an improper experimental method because of unclear boundaries, according to the current results.

Cytotoxicity depends on the time, concentration, and composition of the material [27]. Cell viability increased after treatment with the diluted extracts. Furthermore, cell viability was higher for hardened AH Plus^®^ than that for freshly mixed, as determined by the MTT assay (*p* < 0.05, Figure 1c). It is possible that the extracts from the hardened samples significantly increase cell viability. Freshly mixed AH Plus^®^ decreased cell viability to nearly 0%; however, over time, the cells recovered faster than those treated with other materials [28,29]. Hardened AH Plus^®^ significantly increased cell viability, regardless of the dilution used, suggesting that extracts from the set samples positively affect cell viability. The silicone oil from AH Plus^®^ Paste B may stimulate cell proliferation, as it has been used to decrease glide forces in prefilled syringes [30,31,32]. Cell viability following treatment with Dia-Proseal™ also reached levels greater than 100% for hardened samples, although there were no differences among the dilutions. Freshly mixed AH Plus^®^ and Dia-Proseal™ sealers caused severe cytotoxicity on directly contacting the cells. In contrast, Adseal™ was the most biocompatible material among the three resin-based sealers tested, regardless of whether it was set or unset, with greater than 80% cell viability (the standard for biocompatible dental materials denoted in ISO 7405). It is likely that the iatrogenic release of Adseal™ sealers from the root canal does not result in significant cytotoxic effects. Further, it is possible that including simulated blood flow to the human tooth in these experiments would further alleviate the cytotoxic effects [33,34].

The results of the genotoxicity experiments differed from those of the cytotoxicity assays, which is in agreement with the results of previous studies [35,36]. There are conflicting opinions regarding the genotoxicity of resin-based sealers [36,37,38,39]. Candeiro et al. noted that AH Plus^®^ is significantly more genotoxic than other sealers [40]; however, Van Landuyt et al. concluded that there is no evidence of DNA double-strand breaks caused by the four types of endodontic sealers [35]. The present study investigated resin-based sealers, which showed genotoxicity caused by the release of resin monomers. A critical factor for evaluating genotoxicity using a comet assay is the olive tail moment, which represents tail DNA % by tail moment length. This study showed significant changes in the tail DNA % and tail moment length for all freshly mixed sealers. Furthermore, the tail moment of Adseal™ and AH Plus^®^ significantly increased, regardless of whether they were set or unset, as shown in Figure 2d. Although Adseal™ treatment resulted in the lowest cytotoxicity, Dia-Proseal™ treatment resulted in the lowest genotoxicity, regardless of whether it was set or unset. Therefore, it can be assumed that the genotoxicity does not always coincide with the cytotoxicity result; thus, researchers and clinicians should consider the possibility of the different types of toxicity induced by dental materials before their use.

There are several limitations to this study. First, the international standards for biocompatibility tests of endodontic sealers regulate the in vitro and in vivo tests used; however, there are no experimental models that mimic the human body. The current study is in vitro in nature, in which it primarily focuses on the cytotoxicity and genotoxicity tests performed at a cellular level. Future efforts should include developing a model to evaluate dental materials applied to root canals using a system that accounts for blood flow, which dilutes the toxic effects of the dental materials, such as the in vitro dentin barrier test [41]. Second, the experimental setting in this study shows different usage in terms of the volume and contact area used in the clinic. If the sealers are applied inside the canal, there is a chance that only the material at the apex contacts the periradicular tissues. Therefore, the currently used experimental system of agar overlay, MTT, and comet assay may not reflect the actual clinical conditions. Lastly, some clinicians force the sealers beyond the tooth apex by over-instrumentation. This may damage the underlying tissues and exaggerate the toxicity caused by the canal sealers. Thus, biocompatibility can vary depending on the iatrogenic variables. Considering these limitations, future studies on the biocompatibility of different types of sealers with modified experimental systems are needed.

## 5. Conclusions

Unset epoxy resin-based sealers are generally more cytotoxic and genotoxic than the set materials. Cytotoxicity does not always match the genotoxicity results; therefore, various test tools are required to test toxicity. To establish a biocompatibility test that mimics clinical conditions, it is necessary to properly evaluate the toxic effects.

## Figures and Tables

**Table 1:** Test materials used in this study.

Name	Manufacturer	Materials Group	Setting Time	Lot Number	Composition
Adseal™	Metabiomed, Cheongju-si, Korea	Epoxy resin	50 min	ADS1509221	Base: epoxy oligomer resin, ethylene glycol salicylate, calcium phosphate, bismuth subcarbonate, and zirconium oxide Catalyst: polyaminobenzoate, triethanolamine, calcium phosphate, bismuth subcarbonate, zirconium oxide, and calcium oxide
AH Plus^®^	Dentsply, Charlotte, NC, USA	Epoxy resin	8 h	1511000787	Paste A: epoxy resin, calcium tungstate, zirconium oxide, silica, iron, and oxide pigment Paste B: amine, calcium tungstate, zirconium oxide, silica, and silicone oil
Dia-Proseal™	DiaDent, Cheongju-si, Korea	Epoxy resin	7.5 h	PS15110911118	Base: bisphenol A-co-epichlorohydrin, bisphenol-F epoxy resin, zirconium oxide, silicones, siloxanes, iron oxide, and calcium hydroxide Catalyst: hexamethylenetetramine, zirconium oxide, silicones, siloxanes, calcium hydroxide, and calcium tungstate

**Table 2:** Results of the agar overlay test.

Test Material	Zone Size (cm)	Decolorization Index	Lysis Index	Cell Response	Interpretation
Adseal™ (fresh)	Not defined	5	5	3/3	Severe
Adseal™ (hardened)	0.17 ± 0.11	2	1	2/1	Moderate
AH Plus^®^ (fresh)	Not defined	5	5	3/3	Severe
AH Plus^®^ (hardened)	1.19 ± 0.13	4	5	3/3	Severe
Dia-Proseal™ (fresh)	Not defined	5	5	3/3	Severe
Dia-Proseal™ (hardened)	0.04 ± 0.08	1	0	1/0	Mild
Positive control	0.75 ± 0.08	3	5	2/3	Severe

## Data Availability

The data presented in this study are available on request from the corresponding author.
